# Editorial: Advances in evaluation and management of hair loss disorders

**DOI:** 10.3389/fmed.2023.1131286

**Published:** 2023-01-20

**Authors:** Manuel Valdebran

**Affiliations:** ^1^Department of Dermatology, Medical University of South Carolina, Charleston, SC, United States; ^2^Department of Pediatrics, Medical University of South Carolina, Charleston, SC, United States

**Keywords:** alopecia areata, JAK inhibitor, tofacitinib, corticosteroids, hair loss

Alopecia areata (AA) is a non-scarring hair loss disorder which, depending on the extent of the disease, presents with focal patches of hair loss; complete loss of scalp hair, known as alopecia totalis (AT); or complete loss of scalp, facial and body hair, known as alopecia universalis (AU) (1). Less commonly AA may present as a band-like area on occipitotemporal scalp called ophiasis or sparing temporal and lower occipital scalp called sisaipho (ophiasis spelled backward). There are tools to measure extent of involvement include the “severity of alopecia tool” (SALT) score which is calculated by measuring the percentage of hair loss in each of 3 areas of the scalp: vertex (40%), right profile (18%), left profile (18%), and posterior (24%) (2) and the alopecia density and extent (ALODEX) score which combines both extent and hair density, usually calculated with a tablet (3).

Approximately, 34–50% of patients with AA recover within a year without need of a treatment, however many patients experience relapsing or remitting disease, 10–35% of those progressing to either AT or AU (4).

AA affects 2% of the general population, disproportionately affecting young people with an incidence peaking 20–24 years of age (5); its incidence is higher in females than males. Interestingly, increasing number of cases are seen in certain areas of the globe such as in Central sub-Saharan Africa and Western Sub-Saharan Africa (6).

AA is not a simple cosmetic disease, it is a medical condition with substantial financial burden for patients who must deal with higher plans, pharmacy, and out of pocket expenditures (4). Mesinkovska et al. have shown that patients spend on average $2,000 per year in wigs, hair pieces and psychotherapy visits alone (7). AA decreases health related quality of life, in fact, globally, there is a burden of 7.5 years lost due to AA, with the highest burden in high income North America, followed by Southern Latin America (6). In fact, AA is associated with 70% lifetime prevalence of psychiatric disorders such as anxiety and major depression (8) and many as 78% of adolescents have at least 1 lifetime prevalence of psychiatric disorder (9). Patients consider AA as a daily challenge and burden, with most people rating AA as a moderate or serious burden. As a matter of fact, the burden is significant enough to spend on average 10.3 h per week concealing hair loss (10).

AA is associated with higher incidences of other autoimmune diseases such as thyroid disease, pernicious anemia, and celiac disease and other autoinflammatory disease such as atopic dermatitis, lupus erythematosus, psoriasis, asthma, and allergic rhinitis (6, 11).

Traditionally, therapies to fight this autoinflammatory process have included topical minoxidil, topical contact-sensitizing agents, topical and intralesional corticosteroids and oral medications such as methotrexate or cyclosporine, however, most of the time with incomplete response to treatment, high rate of relapse or limited use due to side effects (12).

In Jun 13, 2022, the FDA approved baricitinib, the first systemic medication for AA, 8 years after Craiglow reported a patient with AU who responded completely to tofacitinib (13). Baricitinib and tofacitinib belong to a pharmacological group called Janus kinase (JAK) inhibitors. JAK is a cytoplasmic tyrosine kinase involved in many different proinflammatory processes within the body (14). Human genetic surveys and gene expression investigations have demonstrated that pathways upstream of the Janus kinases (JAK) are often disrupted in AA (15, 16).

Zhang et al. (17), provide evidence on the effectiveness of oral tofacitinib and systemic corticosteroids alone or combined in patients with moderate-to-severe alopecia areata.

The study has several takeaway points as follows: (1) Non-responding patients to monotherapy alone, achieved SALT 50 after receiving combined therapy (systemic corticosteroids and tofacitinib). (2) All AT and AU patients achieved SALT 50 on combined treatment while 60% did on monotherapy alone. (3) AO subset of patients showed less favorable response to treatment overall (40% in the combined group and 50% in the systemic corticosteroids group).

In addition, investigators report that there were no thromboembolic events, malignancy, or severe infections. The most common infections consisted of nasopharyngitis and upper respiratory infections. Other frequent side effects to be considered were acneiform eruption seen in all groups, as well as weight gain seen in both systemic corticosteroids and combined group.

Lastly, no hepatitis B virus (HBV) reactivation was observed (*n* = 4). Interestingly, the investigation found that two patients in the combined group, experienced elevated HBV DNA levels, however, without liver enzyme abnormalities and liver symptoms.

Although the study comprises small sample, it gives the basis to conduct further studies to elucidate how combined therapies can improve outcomes in AA, such as AU patients non-responding to monotherapy alone as well as characterization of side effects. The substitution of systemic for intralesional corticosteroids combined with JAK inhibitors or the response to newer JAK inhibitors is yet to be elucidated.

## Author contributions

The author confirms being the sole contributor of this work and has approved it for publication.
